# Comparing complications of small-bore chest tubes to large-bore chest tubes in the setting of delayed hemothorax: a retrospective multicenter cohort study

**DOI:** 10.1186/s13049-020-00754-5

**Published:** 2020-06-22

**Authors:** A. Orlando, J. Cordero, M. M Carrick, A. H. Tanner, K. Banton, R. Vogel, M. Lieser, D. Acuna, D. Bar-Or

**Affiliations:** 1 St. Anthony Hospital, Lakewood, CO USA; 2 Swedish Medical Center, Englewood, CO USA; 3 Medical City Plano, Plano, TX USA; 4 Penrose Hospital, Colorado Springs, Colorado Springs, CO USA; 5 Research Medical Center, Kansas City, MO USA; 6 Wesley Medical Center, Wichita, KS USA

**Keywords:** Chest tube, Small-bore, Large-bore, Hemothorax, Complications

## Abstract

**Background:**

Previous studies have provided initial data suggesting that small-bore (SB, ≤ 14Fr) chest tubes have the same efficacy as large-bore (LB, > 14 Fr) chest tubes for acute hemothorax (HTX), but data continue to be lacking in the setting of delayed HTX. This study compared complications of SB chest tubes to LB tubes in patients with delayed HTX.

**Methods:**

This was a retrospective observational study across 7.5 yrs. at 6 Level 1 trauma centers. Patients were included if 1) diagnosed with a HTX or > 1 rib fracture with bloody effusion from chest tube; 2) initial chest tube placed ≥36 h of hospital admission. Patients were excluded for hemopneumothoraces. The primary endpoint was having at least one of the following chest tube complications: tube replacement, VATS, tube falling out, tube clogging, pneumonia, retained HTX, pleural empyema. Secondary outcomes included chest tube output volume and drainage rate. Dependent/independent and parametric/non-parametric analyses were used to assess primary and secondary outcomes.

**Results:**

There were 160 SB patients (191 tubes) and 60 LB patients (72 tubes). Both comparison groups were similar in multiple demographic, injury, clinical features. The median (IQR) tube size for each group was as follows: SB [12 Fr (12–14)] and LB [32 Fr (28–32)]. The risk of having at least one chest tube complication was similar for LB and SB chest tubes (14% vs. 18%, *p* = 0.42). LB tubes had significantly larger risk of VATS, while SB tubes had significantly higher risk of pneumonia. SB tubes had significantly slower least squares (LS) mean initial output drainage rate compared to LB tubes (52.2 vs. 213.4 mL/hour, *p* < 0.001), but a non-parametric analysis suggested no significant difference in median drainage rates between groups 39.7 [23.5–242.0] mL/hr. vs. 38.6 [27.5–53.8], *p* = 0.81. LB and SB groups had similar initial output volume (738.0 mL vs. 810.9, *p* = 0.59).

**Conclusions:**

There was no clearly superior chest tube diameter size; both chest tube sizes demonstrated risks and benefits. Clinicians must be aware of these potential tradeoffs when deciding on the diameter of chest tube for the treatment of delayed HTXs.

## Background

With 300,000 cases annually and causing nearly 25% of deaths in the US trauma population, thoracic trauma is a frequent and serious condition [1, 2]. Thoracic trauma can result in acute hemothoraces (HTXs), and due to their life-threatening nature, nearly all will require an emergent tube thoracostomy. On the other hand, delayed HTXs present later, occurring in 7–12% of thoracic trauma patients, with risks of chest tube thoracostomy ranging between 80 and 100% [3, 4].

Delayed, or non-emergent HTXs are different from acute HTX in that they can be initially so small they do not warrant intervention, or they can be absent on initial chest computed tomography imaging. Although the standard of care for an acute traumatic HTX is the use of a large-bore (LB) 32–36 French (Fr) chest tube, [5] there is no clear chest tube size prescribed for the management of a delayed HTX. Because medicine has become increasingly palliative, some have questioned the practice of using LB chest tubes for the management of thoracic injuries, including HTXs and penumothoraces [6–15].

Anecdotally, the reluctance to utilize small-bore (SB) chest tubes for the treatment of delayed HTXs is due to the belief that, compared to LB chest tubes, SB tubes fall out more often and do not drain the HTX as quickly. On the other hand, it has been argued that SB chest tubes might be superior to LB tubes through decreased tube failure and patient pain [15–17]. Until recently, there has been little published on the topic, leaving clinicians with minimal evidence to substantiate the safety and efficacy of SB chest tubes for delayed HTXs.

Some studies have capitalized on this gulf of information and provided meaningful information. Niinami and colleagues demonstrated that despite the 28 Fr chest tube having a 9-fold greater draining capacity in the in vitro model compared to the 19 Fr tube, the 19 Fr tube drained equally well to the 28 Fr tube in the in vitro porcine models [18]. In a 7-year study comparing 14 Fr to 32–40 Fr chest tubes for delayed HTXs and hemopneumothoraces, both the failure rates, median initial output, and insertion-related complications were all not significantly different between tube sizes [6]. A similar study compared 28–32 Fr versus 36–40 Fr chest tubes for HTXs and also reported no significant differences in initial output, duration of tube placement, or in the tube complication rates; moreover, the mean pain score was smaller for 28–32 Fr versus 36–40 Fr chest tubes [7]. Notwithstanding the growing literature, there continues to be a lack of studies examining small-bore chest tubes in the setting of delayed HTX. The objective of this study was to describe the safety and efficacy of SB (≤14 Fr) chest tubes to LB (> 14 Fr) chest tubes in the setting of delayed HTXs.

## Methods

### Settings and patient population

This was a retrospective observational cohort study utilizing data from six Level 1 Trauma Centers in the Injury Outcomes Network (www.ionresearch.org). Data were obtained via manual chart abstraction and trauma registries. Based on the 2012 Kulvatunyou et al. study, the current study was powered to detect an estimated difference of 16% between comparison groups in the primary outcome. Based on this sample size calculation, for a comparison of two proportions using Pearson’s Chi-square statistic with a Chi-square approximation with a two-sided significance level of 0.05, a sample size of 82 patients per group would achieve a power of at least 0.80 if the proportion of outcome events between groups is different by 16%.

All patients included were admitted between 1/1/2010 and 06/30/2017, had an age ≥ 18 years, suffered a traumatic HTX (ICD-9: 860.2, 860.3; ICD-10: S27.1XXA) or had multiple rib fractures (ICD-9: 807.0–807.19; ICD-10: S22.31XA, S22.31XB, S22.32XA, S22.32XB, S22.39XA, S22.39XB, S22.41XA, S22.41XB, S22.42XA, S22.42XB, S22.43XA, S22.43XB, S22.49XA, S22.49XB) with bloody effusion (captured in medical records), and had an initial chest tube placed ≥36 h from hospital arrival. The time cutoff of ≥36 h was used to identify delayed HTXs. Chest tubes were excluded if placed for hemopneumothoraces or pneumothoraces. Patients with bilateral chest tubes with different categories of chest tube size on each side were excluded (e.g., SB on left side with LB on right side). This study was approved by each facility’s institutional review board and was granted a waiver of informed consent and HIPAA authorization.

### Comparison groups

The two comparison groups for this study were patients with delayed HTXs who were treated with SB chest tubes, and those treated with LB chest tubes. Small-bore chest tubes were defined as those with a diameter ≤ 14 Fr, while LB chest tubes sizes were those with a diameter > 14 Fr [8]. Defining SB chest tubes by the ≤14 Fr cutoff is arbitrary, and was done to aid in comparing the results of the current study to existing studies [6, 8, 10]. All chest tubes were drained via gravity. Patients with SB chest tubes were primarily contributed by one facility that routinely placed them for the management of delayed HTXs. Patients with LB chest tubes were contributed by the remaining five centers that routinely placed them for delayed HTXs. Theoretically, the exchangeability assumption between the comparison groups in this study should hold because treatment with either SB or LB chest tubes was driven by a difference in geographic location, not due to demographic, clinical, or injury characteristics of each patient.

### Covariates

The following demographic and injury characteristics were examined: sex (male, female), age, injury type (blunt, penetrating), highest chest abbreviated injury scale score (AIS), injury severity scale (ISS, 0–8, 9–15, 16–25, ≥26), emergency department (ED) Glasgow coma scale (GCS; 3–8, 9–12, 13–15), normal ED pulse (60–100 beats/minute), normal ED respiratory rate (12–20 breaths/minute), normal ED body temperature (36.6–37.2 °C), normal ED systolic blood pressure (SBP, 90–120 mmHg), coagulation comorbidity (alcoholism, bleeding disorder, on antithrombotic, or coagulation disorder), bilateral tube placement (y/n), total hospital length of stay (LOS).

The following information was collected for each tube: date and time of tube placement, date and time of tube removal, was patient discharged with chest tube (y/n), was video-assisted thoracic surgery (VATS) used for tube placement (y/n), side of tube placement (left anterior, left posterior, right anterior, right posterior), size of chest tube (French scale), initial output of chest tube (milliliters, mL), date and time initial output was measured, rate of chest tube output (mL/hour), and date and time of all chest-tube-related complications (complication must have occurred after tube placement).

### Outcome variables

The primary outcome for this study was having at least one of the following tube complications: 1) requiring a second chest tube (either replacement of initial chest tube or placement of additional tube); 2) tube falling out of patient; 3) tube clogging (chest tube clogged by tissue or other material, not allowing blood to drain properly); 4) pneumonia; 5) pleural empyema; 6) and retained HTX (persistent heterogeneous fluid collection detected by computed tomography within 14 days of initial chest tube placement and requiring intervention [second tube or VATS]). Each chest tube complication must have occurred after the placement of the chest tube. The secondary outcomes of interest were 1) individual tube complications; 2) volume of initial chest tube output (mL); 3) rate of initial chest tube output (mL/hr); and 4) number of hours with each chest tube inserted. The rate of initial chest tube output was calculated by dividing the volume of initial output by the number of hours between chest tube placement and initial output volume measurement.

### Statistical analysis

Because each patient could contribute more than one tube to the study, this study had two levels of analysis: the patient level and the tube level. Patient demographics and injury characteristics were summarized at the patient level, whereas the tube information and tube outcomes were summarized at the tube level. Patient-level data were analyzed using independent methods, and tube-level data were analyzed using dependent methods, accounting for repeated observations.

Chi-squared tests were used to compare proportions, and Fisher’s exact tests were used when more than 50% of cells had estimated counts less than five. A repeated-measures linear regression model was used to compare the mean time each tube was in place and rate of initial output. These models considered the repeated observations, and least squares (LS) means and standard errors (SE) were reported as adjusted measures of central tendency and variability. For non-normal drain rate data, a non-parametric k-sample equality-of-median test was used to test the hypothesis that SB and LB chest tubes were drawn from populations of tubes with similar median drain rates; Fisher’s exact *p*-values were calculated. Another linear regression model was used to examine mean differences between chest tube groups in the total time each patient was on all chest tube(s).

One SB tube patient was missing tube size and a value of 12 Fr was imputed. The patient’s facility mean and median tube size was 12 Fr. Furthermore, this patient had a total of two chest tubes placed, both by interventional radiology; the first tube was 12 Fr but the second tube was lacking a procedure report for the placement of the second tube and thus the tube size was unknown. The mean, median, and mode for chest tubes placed by interventional radiology at this patient’s facility was 11.5 Fr, 12 Fr, and 12 Fr. Therefore, imputing a tube size of 12 Fr for this chest tube was deemed reasonable with low expectation of misclassification.

The time from tube insertion to output measurement was not precisely recorded for 138 tubes in the electronic medical record (SB = 91, LB = 47), and there were 22 tubes with missing chest tube output volumes. Consequently, there were only 122 *tubes* for which a drainage rate could be calculated; 100 *patients* had complete chest tube drainage data, and 120 had missing drainage data for at least one chest tube. The analysis for drainage characteristics is presented only using those 122 chest tubes with drainage rate information. All statistical tests were two-tailed and had an alpha of 0.05; SAS 9.4 (Cary, NC) was used for all analyses.

## Results

**Fig. 1:**
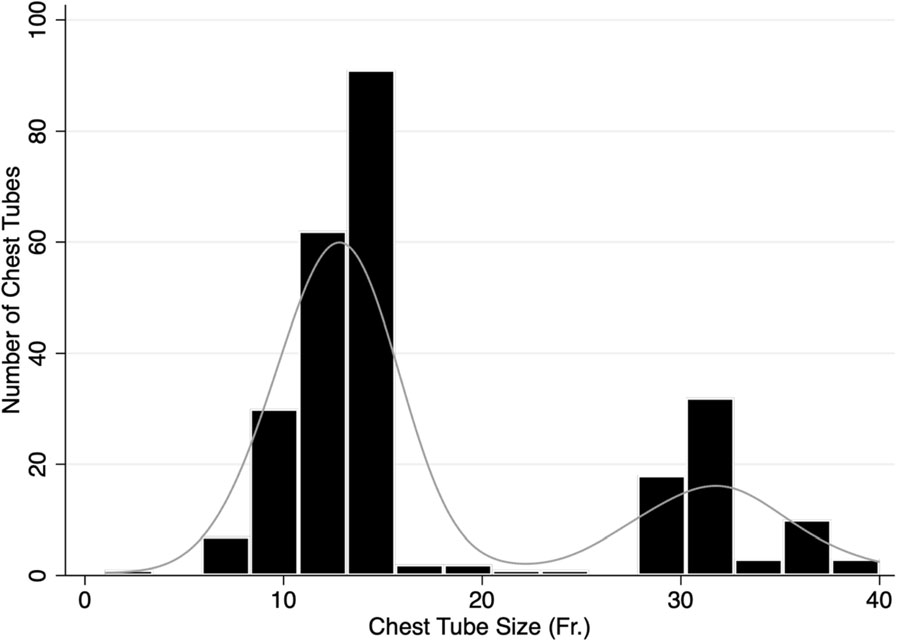
Distribution of chest tube sizes in study population

There was a total of 220 patients included in our study; 160 patients with SB tubes, and 60 with LB tubes. These patients contributed data on 263 chest tubes; 191 SB tubes, and 72 LB tubes. Figure 1 depicts the histogram for chest tube sizes in the study population. The distribution of LB and SB chest tubes were similar based on placement side of chest (Table 1). There were three patients with bilateral chest tubes. Overall, there were 182 (83%) patients contributing a single chest tube, 34 (15%) with two chest tubes, 3 (1%) with three chest tubes, and one patient with four chest tubes (< 1%); there were no significant differences between tube size groups in the total number of tubes contributed by each patient (*p* = 0.38). The median (IQR) tube size for each group was as follows: SB [12 Fr (12-14) and LB [32 Fr (28–32)].

**Table 1:** Demographic and clinical characteristics of 220 patients with delayed hemothorax

Variable, n(%)	Large-Bore(*n* = 60)	Small-Bore (*n* = 160)	P
Male	51 (85%)	112 (70%)	0.02
Age, years			0.67
18 to 29	6 (10%)	13 (8%)	
30 to 49	10 (17%)	38 (24%)	
50 to 69	25 (42%)	69 (43%)	
70 to 89	18 (30%)	36 (23%)	
90+	1 (2%)	4 (3%)	
Injury type			0.11
Blunt	54 (95%)	158 (99%)	
Penetrating	3 (5%)	2 (1%)	
Highest chest AIS value			0.28
< 3	4 (9%)	20 (15%)	
≥ 3	43 (91%)	117 (85%)	
Injury severity scale			0.57
0 to 8	0	3 (2%)	
9 to 15	21 (35%)	68 (43%)	
16 to 25	24 (24%)	53 (33%)	
26+	15 (25%)	35 (22%)	
ED Glasgow coma scale			0.22
3 to 8	8 (16%)	14 (9%)	
9 to 12	2 (4%)	3 (2%)	
13 to 15	41 (80%)	140 (89%)	
ED vital signs ^a^			
Normal pulse	27 (71%)	11 (70%)	0.86
Normal respiratory rate	38 (75%)	121 (79%)	0.55
Normal body temperature	23 (48%)	62 (47%)	0.94
Normal systolic blood pressure	8 (20%)	473 (30%)	0.21
Coagulation comorbidity ^b^	10 (17%)	31 (19%)	0.65
Bilateral chest tubes	1 (2%)	2 (1%)	0.81
Tube placement, side of chest ^c^			0.68
Right	34 (49%)	98 (52%)	
Left	36 (51%)	91 (48%)	
Median (IQR) time from admission until first chest tube, hours ^c^	111 (80–192)	109 (80–176)	0.56

AIS, abbreviated injury scale; ED, emergency department; IQR, interquartile range. Column totals may not add to group total due to missing data

^a^ Normal pulse (60–100 beats per minute); normal respiratory rate (12–20 breaths per minute); normal body temperature (36.6–37.2 C); normal blood pressure (SBP 90–120 mmHg)

^b^ Coagulation comorbidity defined as alcoholism, bleeding disorder, on clopidogrel, coagulation disorder

^c^ Calculated based on each tube

Overall, there were no significant differences between SB and LB patients in demographics, injury severity, or presenting vital signs (Table 1), except for sex; there was a significantly smaller proportion of males in the SB group, than in the LB group. Sex was not considered a potential confounding variable, nor was it identified in any models as an independent predictor of outcomes. After adjustment for repeated measures, there was no significant difference between LB and SB chest tubes in the length of time each tube was in place [130.13 (41.9) hrs vs. 89.49 (24.1) hrs, *p* = 0.40]. Table 2 shows where each chest tube was placed and the staff who placed them.

**Table 2:** Chest tube groups and complications, by insertion location and staff

N (%)	Large-Bore	Small-Bore	P	Any Tube Complication	P
	(*n* = 72)	(*n* = 191)		(*n* = 45)	
Location of tube placement			< 0.001		0.91
Bedside	32 (44%)	86 (45%)		19 (16%)	
Interventional radiology	6 (8%)	100 (52%)		19 (18%)	
Operating room	34 (47%)	4 (2%)		7 (8%)	
Staff who placed tube			0.10		0.20
Physician	51 (71%)	137 (72%)		36 (19%)	
Surgical resident	2 (3%)	18 (9%)		5 (9%)	
Physician assistant	19 (26%)	36 (19%)		4 (20%)	

An equivalent proportion of SB and LB chest tubes were placed at the bedside. However, SB chest tubes were more often placed in interventional radiology, whereas LB tubes were more often placed in the operating room (Table 2). Additionally, there were similar proportions of SB and LB chest tubes placed by physicians, surgical residents, and physician assistants. Each patient had an LS mean (SE) total time on chest tubes of 144 (50) hours in the LB group, and 87 (29) hours in the SB group; this difference was not statistically significant (Table 3).

**Table 3:** Tube and patient outcomes by comparison groups

Variable, n (%)	Large-Bore	Small-Bore	P
**Tube outcomes**	**n = 72**	**n = 191**	
≥ 1 tube complication	10 (14%)	35 (18%)	0.42
Tube replaced	6 (8%)	8 (4%)	0.22
VATS	4 (6%)	0	0.005
Tube fell out	0	7 (4%)	0.20
Tube clogged	1 (1%)	8 (4%)	0.45
Pneumonia	0	14 (7%)	0.01
Pleural empyema	1 (1%)	2 (1%)	> 0.99
Retained hemothorax	3 (4%)	5 (3%)	0.69
Drainage metrics	*n* = 22	*n* = 100	
Time from chest tube insertion until initial output measurement, LS mean (SE), hr	12.8 (1.75)	20.6 (0.86)	< 0.001
Initial output, LS mean (SE), mL	738.0 (121.56)	810.9 (57.02)	0.59
Rate of initial output, LS mean (SE), mL/hr	213.4 (32.98)	52.2 (15.47)	< 0.001
**Patient outcomes**	**n = 60**	**n = 160**	
Total time on chest tube(s), LS mean (SE), hr	144.6 (50.05)	86.5 (28.80)	0.32
Hospital LOS, LS mean (SE), day	15.0 (1.26)	15.1 (0.73)	0.95
Patient returned to prior lung function ^a^	25 (42%)	42 (36%)	0.43

VATS, video assisted thoracic surgery; CI, confidence interval; LS, least squares; SE, standard error; IQR, interquartile range; mL, milliliters; LOS, length of stay

^a^ Assessed at discharge by physical therapy; 42 small-bore patients missing data

Seventeen percent of tubes suffered at least one complication. The results of the primary outcome analysis suggested no significant difference between chest tube groups in the risk of having at least one tube complication (Table 3). The major significant difference in tube-related complications between chest tube groups were due to VATS and pneumonia events (Table 3). Although LB chest tubes carried a significantly higher risk of subsequent VATS, SB chest tubes had a higher risk of pneumonia. The size of tubes that subsequently required VATS were 32, 32, 32, and 34 Fr. Meanwhile, the median (IQR) size of tubes associated with pneumonia was 14 Fr (12-14). These study data did not indicate there were significant differences between SB and LB chest tubes in the risk of tubes requiring replacement, falling out, or clogging; or resulting in pleural empyema or retained HTX.

There were notable differences in drainage characteristics between the two tube groups (Table 3). Volume of initial output for SB chest tubes was assessed an average of 7.8 h later from tube insertion, compared to LB chest tubes (*p* < 0.001). There was no significant difference in the mean initial output volume between LB and SB chest tubes. Ultimately, LB chest tubes had a drainage rate that was 4-fold faster than SB chest tubes (213.4 mL/hr. vs. 52.2 mL/hr., p < 0.001). According to a non-parametric test of medians, there was no difference between LB and SB chest tubes in the median (IQR) drainage rates (39.7 [23.5–242.0] mL/hr. vs. 38.6 [27.5–53.8], *p* = 0.81). Demographic and clinical characteristics were compared between patients with complete chest tube drainage information and those missing drainage data (Supplemental Table S1). Patients with complete data had significantly lower proportions of patients aged 18–29 years, and higher proportions of patients aged 70–89 years. Additionally, patients with complete drainage data were less severely injured according to the injury severity scale. Lastly, there was a higher proportion of patients with complete data who had normal body temperature at admission, compared to patients with missing drainage data.

Nevertheless, both tube size groups had similar mean total lengths of time with chest tubes throughout their hospitalization. Finally, there were no significant differences between chest tube groups in LS mean hospital LOS nor in the proportion of patients who returned to prior lung function.

## Discussion

In the setting of delayed HTXs, this is one of the first studies to include an examination of SB chest tubes smaller than 14 Fr [6–8, 11, 15] and contributes the history of incremental reporting of increasingly smaller chest tube diameters. According to the data, there were no significant differences between chest tubes ≤14 Fr vs. > 14 Fr in the probability of experiencing at least one chest tube complication (Table 3). Furthermore, there were no major differences between groups in the drainage rate or volume, though drainage rate data should be interpreted with caution.

The current study used a different primary outcome compared to previous studies, with the aim of providing a more global perspective on chest tube outcomes, [6, 8, 13] and most closely resembles the primary outcome presented in the Tanizaki et al. study [11]. Previously, tube-related outcomes have been separated into failures (e.g. VATS, requiring a second tube) and complications (e.g. tube fell out, clogging of tube, retained HTX, pneumonia, pleural empyema). Instead, the current study considered each of these outcomes a tube complication. We believe this allowed for an overall comparison of risk of complication between SB and LB chest tubes. Despite the difference in definition of the primary endpoint in the current study, these results were consistent with multiple studies examining the use of SB chest tubes for the management of HTXs, particularly with respect to the higher risk of VATS with the use of LB chest tubes [6, 8, 11].

On the contrary, unlike previously published studies on the topic, the current study documented an increased risk of pneumonia with the use of SB chest tubes in the setting of delayed HTXs [6–8, 11, 19]. It is possible that the development of pneumonia could have been precipitated by the development of another or previous complication, including a retained HTX or pleural empyema. However, of the 14 patients who developed pneumonia, only two suffered another tube complication: one patient had a tube replaced, and the second patient’s chest tube fell out. Therefore, there was no clear link between the development of pneumonia and a previous or concomitant complication. A possible cause of the increased risk of pneumonia could be related to the number of rib fractures or ventilator days [20, 21]; however, neither were available for data analysis. Furthermore, due to limitations in drainage rate data and the fact that all pneumonia events occurred in the SB chest tube group, we could not investigate the relationship between chest tube drainage rate and the development of pneumonia. It is also possible this finding is due to chance alone. This potentially serious risk must be weighed against the observed benefits of SB chest tubes.

Anecdotally, LB chest tubes are preferred to SB tubes primarily because they are thought to more readily drain a HTX. The current study examined the initial output volume and rate of initial output for each chest tube. According to the current study, LB chest tubes were not significantly different from SB chest tubes regarding the volume of initial output (Table 3); this finding is consistent across the literature on thoracic trauma [6–8, 10, 11, 14]. According to theory, a 28 Fr chest tube should have roughly 13-fold the draining capacity of a 14 Fr chest tube [18]; the current study data contradict this notion. Caution is necessary when interpreting the tube drainage rate information, as it comes from a subset (*n* = 122) of the larger study population. Additionally, a lack of consistency in measuring time from chest tube insertion to output volume measurement likely caused inaccurate estimations of the drainage rates, likely secondary to inaccurate times in the denominators of each rates. This is plausible given the lack of control in data collection with a retrospective study. Nevertheless, a slower drainage rate by SB chest tubes might not preclude their use in the setting of delayed HTX. Slow pleural drainage rates are likely to be problematic in the setting of acute traumatic HTXs which require rapid evacuation of pleural blood. But, in the setting of delayed or non-emergent HTXs, the slower drainage of SB chest tubes might be an acceptable tradeoff given their potential benefits vis-à-vis lowered pain [15, 17].

The current study had lower power to detect differences between chest tube groups in individual complications. However, an absence of evidence is not evidence of absence, making it important to consider non-significant results. Despite non-significance, SB tubes had a nominal 4-fold higher risk of falling out and clogging when compared to their LB counterparts. This is supported by other studies documenting increased risks of SB chest tubes clogging and falling out [12, 17]. It therefore remains important to consider these risks and implement mitigating strategies to prevent their occurrence when using SB chest tubes. These include the development of novel techniques for securing chest tubes to the chest wall, and the frequent flushing of SB chest tubes with saline [12].

It is necessary to consider the limitations of this study. First, the retrospective non-randomized nature of this study limits its generalizability and proximity to elucidating causal associations. One facility in this multi-center collaboration routinely placed SB chest tubes for delayed HTXs, while the other five centers routinely placed LB chest tubes. Owing to the lack of differences between chest tube size groups in demographic, clinical, and injury characteristics, we believe these results are not heavily influenced by selection bias between the study hospitals; nevertheless, it cannot be completely dismissed. Because the success of a chest tube is dictated by correct placement, the current study is favorable because it included the practice variability for LB chest tube placement across five Level I Trauma Centers; however, there is less practice variability in the placement of the SB chest tubes as they were placed primarily at a single hospital. Despite the non-randomized nature of this study, both study groups were similar in terms of demographics, overall injury characteristics, the mean time from admission until first chest tube placement, and the mean duration with a chest tube. The lack of treatment randomization allows for residual confounding and unmeasured confounding to be possible contributors to the results in the current study. Furthermore, due to the limitations of patient chart review, the amount of time that lapsed between chest tube placement and chest tube output measurement was not precisely recorded for a majority of patients in this study, and results from the analysis of mean drainage rates should not be over interpreted; general conclusions should be drawn from the non-parametric comparison of median drainage rates. Owing to the limitations inherent with retrospective cohort studies, these results should be confirmed in a randomized clinical trial. Accurately assessing drainage rates is vital to comparing the performance of SB and LB chest tubes and should be consistently measured in future prospective observational and randomized study designs.

## Conclusions

The current study described the complication and drainage characteristics of SB and LB chest tubes in the management of delayed HTXs. Compared to LB chest tubes, there was no clear overall benefit or harm to the use of SB in the setting of delayed HTXs. Because of the continued equipoise regarding the use of SB versus LB chest tubes, there remains a need for a randomized clinical trial. If SB chest tubes are used to treat delayed HTXs, physicians must be vigilant to the development of pneumonia and the potential for the chest tubes to become dislodged or clogged.

## Supplementary information

**Additional file 1: Table S1.** Comparison of demographic and clinical characteristics between patients with and without missing chest tube drainage data

## Data Availability

The datasets used and/or analyzed during the current study are available from the corresponding author on reasonable request.

## Abbreviations

HTX: Hemothorax
LB: Large-bore
SB: Small-bore
Fr: French
ICD: International classification of disease
AIS: Abbreviated injury scale
ISS: Injury severity scale
ED: Emergency department
GCS: Glasgow coma scale
C: Celsius
SBP: Systolic blood pressure
VATS: Video-assisted thoracic surgery
mL: Milliliters
LS: Least squares
SE: Standard error
NC: North Carolina (USA)
HIPAA: Heath Insurance Portability and Accountability Act
IQR: Interquartile range
LOS: Length of stay
