# Supplementary material for: Comparing complications of small-bore chest tubes to large-bore chest tubes in the setting of delayed hemothorax: a retrospective multicenter cohort study
Source: Scand J Trauma Resusc Emerg Med. 2020 Jun 22;28:56. doi: 10.1186/s13049-020-00754-5 (PMC7310264; doi:10.1186/s13049-020-00754-5)
Supplement: Supplementary file 1 — Additional file 1: Table S1. Comparison of demographic and clinical characteristics between patients with and without missing chest tube drainage data [file 13049_2020_754_MOESM1_ESM.docx]

**SUPPLEMENTAL TABLE S1**

| **Table S1.** Comparison of demographic and clinical characteristics between patients with and without missing chest tube drainage data. | | | | |
| --- | --- | --- | --- | --- |
| Variable, n(%) | **No Missing  Drainage Data** N=100 | **Missing  Drainage Data** N=120 | **P** |  |
| Male | 71 (71%) | 92 (77%) | 0.34 |  |
| Age, years |  |  | 0.03 |  |
| 18 to 29 | 3 (3%) | 16 (13%) |  |  |
| 30 to 49 | 21 (21%) | 27 (23%) |  |  |
| 50 to 69 | 42 (42%) | 52 (43%) |  |  |
| 70 to 89 | 32 (32%) | 22 (18%) |  |  |
| 90+ | 2 (2%) | 3 (3%) |  |  |
| Injury type |  |  | >0.99 |  |
| Blunt | 97 (98%) | 115 (97%) |  |  |
| Penetrating | 2 (2%) | 3 (3%) |  |  |
| Highest chest AIS value |  |  | 0.18 |  |
| < 3 | 60 (82%) | 100 (90%) |  |  |
| ≥ 3 | 13 (18%) | 11 (10%) |  |  |
| Injury severity scale |  |  | 0.01 |  |
| 0 to 8 | 3 (3%) | 0 |  |  |
| 9 to 15 | 48 (48%) | 41 (34%) |  |  |
| 16 to 25 | 33 (33%) | 44 (37%) |  |  |
| 26+ | 15 (15%) | 35 (29%) |  |  |
| ED Glasgow coma scale |  |  | 0.30 |  |
| 3 to 8 | 7 (7%) | 15 (13%) |  |  |
| 9 to 12 | 3 (3%) | 2 (2%) |  |  |
| 13 to 15 | 86 (90%) | 95 (85%) |  |  |
| ED vital signs^a^ |  |  |  |  |
| Normal pulse | 70 (77%) | 67 (64%) | 0.06 |  |
| Normal respiratory rate | 74 (76%) | 85 (79%) | 0.68 |  |
| Normal body temperature | 46 (55%) | 39 (41%) | 0.05 |  |
| Normal systolic blood pressure | 19 (22%) | 36 (33%) | 0.08 |  |
| Coagulation comorbidity^b^ | 14 (14%) | 28 (23%) | 0.07 |  |
| AIS, abbreviated injury scale; ED, emergency department; IQR, interquartile range. | | | | |
| ^a^ Normal pulse (60-100 beats per minute); normal respiratory rate (12-20 breaths per minute); normal body temperature (36.6-37.2 Celsius); normal blood pressure (SBP<120 mmHg). | | | | |
| ^b^ Coagulation comorbidity defined as alcoholism, bleeding disorder, on clopidogrel, coagulation disorder. | | | | |
